# Dnmt1 associated *Gja1* promoter methylation changes are implicated in Cx43 remodeling during acute myocardial ischemia/reperfusion injury

**DOI:** 10.1080/15592294.2026.2694817

**Published:** 2026-07-01

**Authors:** Yang Liu, Zijun Wang, Youxiao Zhao, Yanyan Ma, Jing Yi, Yingnan Song, Yanqiu Liu, Li An, Zhijun Pan, Hong Gao, Ying Cao

**Affiliations:** a Department of Anesthesiology, The Affiliated Hospital of Guizhou Medical University, Guiyang, Guizhou, China; b Department of Anesthesiology, The Affiliated Jinyang Hospital of Guizhou Medical University, The Second People’s Hospital of Guiyang, Guiyang, Guizhou, China; c School of Anesthesiology, Guizhou Medical University, Guiyang, Guizhou, China; d Department of Anesthesiology, The First People’s Hospital of Zunyi, Zunyi, Guizhou, China; e Guizhou Medical University Key Laboratory of Cardiovascular Disease Basic and Clinical Research, Department of Physiology, School of Basic Medical Sciences, Guizhou Medical University, Guiyang, Guizhou, China; f Department of Anesthesiology, The Fourth People’s Hospital of Guiyang, Guiyang, Guizhou, China; g Department of Anesthesiology, Guiyang Maternal and Child Health Care Hospital and Guiyang Children’s Hospital, Guiyang, Guizhou, China

**Keywords:** DNA methylation, Dnmt1, connexin 43 remodeling, myocardial ischemia/reperfusion injury, MIRI, epigenetic regulation

## Abstract

DNA methylation has traditionally been regarded as a stable epigenetic modification. However, emerging evidence indicates that oxidative and genotoxic stress can induce locus-specific methylation changes that modulate transcriptional responses. The contribution of such dynamic regulation to cardiac gene expression during myocardial ischemia/reperfusion injury (MIRI) remains incompletely understood. This study investigated whether altered methylation changes within the *Gja1* promoter region are associated with connexin43 (Cx43) remodeling during MIRI. Male C57BL/6 mice were underwent global hypothermic I/R using a Langendorff-perfused heart model, with or without pretreatment using the DNA methyltransferase inhibitor 5-azacytidine (5-Aza, 3 μg/g body weight, i.p.). *Gja1* promoter methylation was analyzed by BSP and MSP, while Dnmt1 enrichment was assessed by chromatin immunoprecipitation. Cx43 expression and localization were examined by qRT-PCR, Western blotting, and immunofluorescence, together with electrophysiological and histological analyses. MIRI was associated with Dnmt1 upregulation and nuclear enrichment, accompanied by altered methylation signals at selected CpG sites within the examined *Gja1* promoter region and transcriptional repression. These changes coincided with reduced Cx43 expression, altered subcellular localization, impaired electrical conduction and increased arrhythmia susceptibility. Pretreatment with 5-Aza partially attenuated *Gja1* promoter methylation signals and preserved Cx43 expression and localization during subsequent MIRI. These findings support an association between Dnmt1 enrichment, altered*Gja1*promoter CpG methylation, and Cx43 remodeling under reperfusion stress, suggesting that methylation-related epigenetic regulation may participate in Gja1 transcriptional responses during MIRI. Further temporal and gene-specific validation will be required to establish the kinetics and causal specificity of this pathway.

## Introduction

Advances in anesthesia, cardiac surgery, and extracorporeal circulation have established surgical intervention as a cornerstone therapy for severe cardiovascular diseases. However, cardiac procedures utilizing cardiopulmonary bypass (CPB) necessitate aortic cross-clamping, which completely interrupts coronary blood flow and subjects the myocardium to ischemia. Although cardioprotective strategies including hypothermic preservation and high-potassium cardioplegic arrest are routinely employed, reperfusion arrhythmias (RAs) and myocardial stunning remain prevalent complications following aortic declamping and restoration of coronary circulation [1–3]. Current research on RAs is predominantly focused on medical treatments, including the identification of risk factors and the mechanisms underlying coronary reperfusion procedures, with limited mechanistic understanding of the molecular pathways governing arrhythmogenesis during CPB-associated ischemia/reperfusion (I/R) [4,5]. Consequently, there is a significant need to explore the mechanisms of RA and to develop preventive measures, which would greatly improve patient prognosis.

Connexin 43 (Cx43) serves as the predominant gap junction protein in ventricular myocardium, forming intercellular channels essential for synchronized electrical conduction and coordinated cardiac contraction [6]. Normal cardiac electrophysiology depends critically on the precise regulation of Cx43 expression, post-translational modifications, and subcellular localization [7]. Pathological alterations in Cx43 including decreased expression, aberrant phosphorylation, or redistribution from intercalated discs have been causally linked to diverse arrhythmic disorders, encompassing atrial fibrillation, ventricular tachyarrhythmias, and reperfusion-induced conduction disturbances. These molecular perturbations manifest functionally as impaired impulse propagation, enhanced arrhythmia susceptibility, and adverse cardiac remodeling [8,9]. While therapeutic modulation of Cx43 through pharmacological agents, gene delivery systems, and conditioning protocols has demonstrated cardioprotective efficacy [10–12], the upstream regulatory mechanisms controlling Cx43 expression during I/R injury remain incompletely characterized.

DNA methyltransferases (DNMTs) constitute a family of epigenetic enzymes that catalyze cytosine methylation at CpG dinucleotides, thereby modulating gene transcription without altering DNA sequence [13,14]. The DNMT family comprises two functionally distinct subfamilies: de novo methyltransferases (DNMT3A and DNMT3B) that establish methylation patterns during development and differentiation, and the maintenance methyltransferase DNMT1, which preserves existing methylation marks through DNA replication cycles [15,16]. DNMT1 can be rapidly recruited to chromatin in response to oxidative stress and DNA damage, independent of DNA replication. Specifically, reactive oxygen species (ROS) as a hallmark of myocardial ischemia/reperfusion injury (MIRI) have been shown to trigger the formation of a silencing complex containing DNMT1 and SIRT1 at GC-rich promoter regions, leading to acute focal hypermethylation and transcriptional silencing within minutes to hours of injury [17,18]. While emerging evidence implicates aberrant DNMT1 activity in cardiovascular pathophysiology, such as drug-induced cardiotoxicity and cardiac fibrosis [18–20]. However, the potential regulatory relationship between DNMT1 and Cx43 expression, and its contribution to I/R-induced arrhythmogenesis remains largely unexplored. Although previous studies have established correlations between Cx43 dysregulation and reperfusion arrhythmias, the specific epigenetic mechanisms underlying Cx43 transcriptional control during I/R have not been elucidated.

In the present study, we investigated whether I/R stress is associated with DNMT1-related epigenetic regulation of *Gja1* transcription. Using a Langendorff-perfused mouse heart model combined with molecular and biochemical analyses, we examined changes in Dnmt1 expression, promoter methylation status of *Gja1*, and connexin43 remodeling during I/R. In addition, we assessed whether pharmacological modulation of DNA methylation could partially reverse these molecular alterations. This study aims to provide mechanistic insight into the epigenetic regulation of gap junction gene expression under pathological stress conditions.

## Material and methods

### Animals

Male C57BL/6 mice (aged 8–10 weeks, weighing 20–24 g) were obtained from the Experimental Animal Center of Guizhou Medical University. The mice were housed at 23°C and 60% humidity on a 12/12 h light/dark cycle with free access to food and water. Only male mice were used in this initial mechanistic study to maintain a relatively homogeneous experimental background, the present study was not designed or powered to evaluate sex-specific differences. All experimental protocols adhered to institutional animal welfare guidelines and received approval from the Institutional Animal Ethics Committee of The Third Affiliated Hospital, Guizhou Medical University (approval number: 2020A008).

### In vivo administration of DNMT inhibitor

The DNA methyltransferase inhibitor group was treated with intraperitoneal injections of 100 μl 5-Azacytidine (5-Aza, 3 μg/g dissolved in 0.9% normal saline; Abcam, Cambridge, UK) on days 8, 9, 10, 12, and 14, while the control group received saline. On day 14, directly after the final injection, all mice were subjected to Langendorff isolated heart perfusion model establishment and intervention procedures.

### Preparation of Langendorff-perfused isolated hearts

Following a 6-h fasting period, mice (10–12 weeks old, 26–30 g) received systemic anticoagulation with heparin sodium (5 IU/g, intraperitoneal) followed by sodium pentobarbital anesthesia (50 mg/kg, intraperitoneal) after a 15-min interval. In accordance with the American Veterinary Medical Association (AVMA) Guidelines for the Euthanasia of Animals, deep anesthesia was confirmed by loss of the righting reflex and absence of pedal withdrawal. Cardiac excision was performed via midline thoracotomy, and hearts were immediately immersed in ice-cold modified Tyrode’s solution containing (in mM): NaCl 119, KCl 4.0, MgCl_2_·6H_2_O 1.0, NaHCO_3_ 25, KH_2_PO_4_ 1.2, CaCl_2_ 1.8, and glucose 10 (pH adjusted to 7.35–7.45).

Hearts were cannulated via the ascending aorta and mounted on a Langendorff perfusion apparatus (IH-SR system, Hugo Sachs Elektronik, March-Hugstetten, Germany) for retrograde coronary perfusion. The perfusion medium consisted of oxygenated Tyrode’s solution (95% O_2_/5% CO_2_) delivered at constant pressure (60 mmHg), flow rate (2–4 mL/min), and temperature (37°C). Hearts were included if successful cannulation and stable retrograde perfusion were achieved and spontaneous sinus rhythm with a heart rate >300 beats per minute was restored within 5 min of reperfusion. Exclusion criteria included unsuccessful cannulation, unstable perfusion, or sustained arrhythmias during equilibration. Inclusion and exclusion criteria were predefined prior to the experiments, and all isolated hearts fulfilled the inclusion criteria in the present study.

### Establishment of hypothermic global ischemia/reperfusion model

After 15 min of equilibration perfusion, hearts were randomly allocated using computer-generated numbers to four experimental groups (*n* = 8 per group): saline control, saline ischemia/reperfusion (saline I/R), 5-Aza control, and 5-Aza ischemia/reperfusion (5-Aza I/R). Control groups underwent continuous perfusion with 37°C Tyrode’s solution for 120-min total. The ischemia/reperfusion groups, simulating clinical cardioplegic arrest conditions: following 30 min of baseline perfusion, global ischemia was induced by switching to hypothermic (4°C) St Thomas’ cardioplegic solution containing (in mM): NaCl 119, KCl 20, MgCl_2_·6 H_2_O 16, NaHCO_3_ 25, CaCl_2_ 1.8 (pH 7.8). Initial cardioplegia delivery (20 ml/mg) through the aortic root connector of the IH-SR system to induce cardiac arrest for 60 min (with an additional half dose of 4°C St Thomas’ cardioplegic solution, 10 ml/mg, administered at 30 min into the arrest period), followed by 30 min of reperfusion with 37°C Tyrode’s solution. During the arrest period, the heart was protected with 4°C St Thomas’ cardioplegic solution.

### Electrophysiological assessment

Cardiac electrical activity was monitored at four predetermined intervals: baseline equilibration (T0, 15 min), pre-intervention (T1, 30 min), mid-protocol assessment (T2, corresponding to 95 min continuous perfusion for control groups or 5 min post-reperfusion for I/R groups), and terminal assessment (T3, corresponding to 120 min continuous perfusion for control groups or 30 min post-reperfusion for I/R groups). Standard electrocardiographic recording utilized bipolar electrodes positioned on the right atrium and left ventricular apex. Concurrently, high-resolution epicardial mapping was performed using a 64-channel microelectrode array system (EMS64-USB-1003, MappingLab Ltd., Sheffield, UK) positioned on the left ventricular anterior wall. The mapping system captured extracellular field potentials, activation sequences, and arrhythmic events with spatial resolution of 300 μm and temporal resolution of 1 ms. Data acquisition and analysis were performed using EMapRecord 5.0 software, enabling calculation of local conduction velocity (CV) from activation time differences between adjacent electrodes and quantification of conduction heterogeneity (CHI) through coefficient of variation analysis.

### Histopathological evaluation

Following electrophysiological studies, cardiac tissue was rapidly harvested and cardiac apex specimens were immersion-fixed in 4% buffered paraformaldehyde for 48 h. After routine dehydration and paraffin embedding, 5 μm sections were prepared and stained with hematoxylin and eosin using standard protocols. Histological assessment was performed using a digital slide scanner (10×, 40×, VS200, Olympus Life Science, Japan).

### Apoptosis detection

Cardiomyocyte apoptosis was evaluated using terminal deoxynucleotidyl transferase-mediated dUTP nick-end labeling (TUNEL) according to manufacturer specifications (One-step TUNEL In Situ Apoptosis Kit, Green, FITC, E-CK-A320. Elabscience Biotechnology, Wuhan, China). Deparaffinized sections underwent antigen retrieval, followed by sequential incubation with DNase I buffer, TdT equilibration buffer, and TdT reaction mixture. Nuclear counterstaining was achieved with DAPI. Fluorescence images were captured using an inverted microscope (IX83, Olympus Life Science, Japan) with sequential scanning at 405 nm and 488 nm (40× magnification). Apoptotic index was calculated as the percentage of TUNEL-positive nuclei relative to total DAPI-stained nuclei across 10 randomly selected high-power fields.

### Molecular analysis

#### RNA extraction and quantitative RT-PCR (qRT-PCR)

Total RNA was extracted from left ventricular myocardial tissue using TRIzol reagent (Thermo Fisher Scientific, Waltham, MA) following manufacturer protocols. RNA integrity was verified by agarose gel electrophoresis, and concentration was determined spectrophotometrically. Complementary DNA synthesis was performed using a reverse transcription system (Vazyme Biotech, Nanjing, China). qPCR was performed using the primers listed in the Supplementary Table S1. Target genes were normalized to GAPDH expression, and the relative expression levels were determined using the 2-△△CT method.

#### Protein expression analysis

Ventricular tissue samples were mechanically homogenized in RIPA extraction buffer supplemented with protease and phosphatase inhibitors. Following centrifugation at 12,000 × g for 15 min at 4°C, protein concentrations in supernatants were determined using the Bradford assay. Equal amounts of denatured protein (30 μg) were resolved by SDS-polyacrylamide gel electrophoresis and electrotransferred to polyvinylidene fluoride membranes. After blocking with 5% bovine serum albumin, membranes were probed overnight at 4°C with primary antibodies, followed by horseradish peroxidase-conjugated secondary antibodies. Immunoreactive bands were visualized using enhanced chemiluminescence detection and quantified by densitometric analysis (ImageJ software, National Institutes of Health).

#### Immunofluorescence

Paraffin-embedded sections underwent heat-induced antigen retrieval in citrate buffer (pH 6.0) followed by permeabilization and blocking with 5% normal serum. Primary antibodies against Cx43 (1:2000 dilution, Sigma-Aldrich) and Dnmt1 (1:1000 dilution, Abcam) were applied overnight at 4°C. Detection was accomplished using species-appropriate fluorophore-conjugated secondary antibodies (FITC anti-rabbit 1:2000, CY3 anti-mouse 1:1000, Boster Biological Technology). Nuclear counterstaining utilized DAPI. Fluorescence intensity measurements were performed across 10 randomly selected fields per section using ImageJ analysis software.

#### DNA methylation analysis

Genomic DNA was extracted from mouse cardiac tissue using a commercial extraction kit (ELK Biotechnology, Wuhan, China) according to the manufacturer’s protocol. Bisulfite conversion was performed using the EZ DNA Methylation-Gold Kit (Zymo Research, Irvine, CA, USA) to convert unmethylated cytosines to uracil while preserving methylated cytosines. Primers for bisulfite sequencing PCR (BSP) and methylation-specific PCR (MSP) were designed using MethPrimer based on CpG island prediction within the *Gja1* (Cx43) promoter region, avoiding CpG sites within primer-binding regions. For clone-based BSP analysis, bisulfite-treated DNA was amplified using promoter-specific primers (Supplementary Table S2.1), and PCR products were cloned into pGEM-T Easy vectors (Promega, Madison, WI, USA). A 472 bp *Gja1* promoter fragment containing eight CpG dinucleotides was analyzed. For each animal, approximately 10 independent positive clones were selected for Sanger sequencing. CpG methylation status was determined based on cytosine retention after bisulfite conversion and analyzed using BiQ Analyzer. Clone-level and per-CpG methylation patterns were visualized using lollipop plots, with filled circles indicating methylated CpGs and open circles indicating unmethylated CpGs. Overall methylation levels were calculated as the percentage of methylated CpG sites across all analyzed clones. For MSP analysis, bisulfite-treated DNA was amplified using methylated (M) and unmethylated (U) primer sets targeting the Cx43 promoter (Supplementary Table S2.2). PCR products were separated on 2% agarose gels and visualized under UV illumination. The presence of M or U bands indicated the corresponding methylation status of the Cx43 promoter.

#### Chromatin immunoprecipitation (ChIP)

Cross-linking of protein-DNA complexes was achieved by treating cardiac tissue with formaldehyde (1% final concentration) for 20 min at room temperature, followed by quenching with glycine (0.125 M final concentration) to terminate the cross-linking reaction. Chromatin was extracted using lysis buffer supplemented with protease inhibitors and subsequently fragmented to 200–400 base pairs by sonication. Fragment size distribution was verified by agarose gel electrophoresis to ensure optimal shearing efficiency. Immunoprecipitation was performed overnight at 4°C using Dnmt1-specific antibodies (2 μg per 25 μg chromatin) or matched control IgG as a negative control. Immune complexes were captured using protein A-agarose beads and subjected to sequential washing with buffers of increasing stringency to remove non-specific binding. Cross-links were reversed, and DNA was recovered through phenol-chloroform extraction followed by ethanol precipitation. Quantitative PCR analysis was performed on the immunoprecipitated DNA using primers specifically designed for the Cx43 promoter region (primer sequences provided in Supplementary Table S2). The enrichment of Dnmt1 binding at the Cx43 promoter was calculated relative to input DNA and normalized against IgG control values.

## Statistical analysis

All data are expressed as the mean ± standard deviation (SD) from at least six independent biological replicates. Investigators conducting the experiments were aware of group allocation, whereas outcome assessment and data analysis were performed using predefined objective criteria and coded datasets to minimise bias. Statistical analyses were performed using GraphPad Prism version 9.0 (GraphPad Software, La Jolla, CA, USA) and SPSS version 26.0 (IBM Corp., Chicago, IL, USA). For continuous variables (e.g., body weight, conduction velocity, heart rate, and time to rhythm resumption), group comparisons were conducted using one-way analysis of variance (ANOVA) followed by Tukey’s multiple comparison test when appropriate. In cases involving two independent groups, Student’s t test was applied. For categorical variables (e.g., incidence of arrhythmias and fatal arrhythmias), Fisher’s exact test was employed due to the small sample size (*n* = 8 per group). A two-sided *p* value <0.05 was considered statistically significant for all analyses.

## Results

### DNMT inhibition reduces RAs

**Figure 1.**
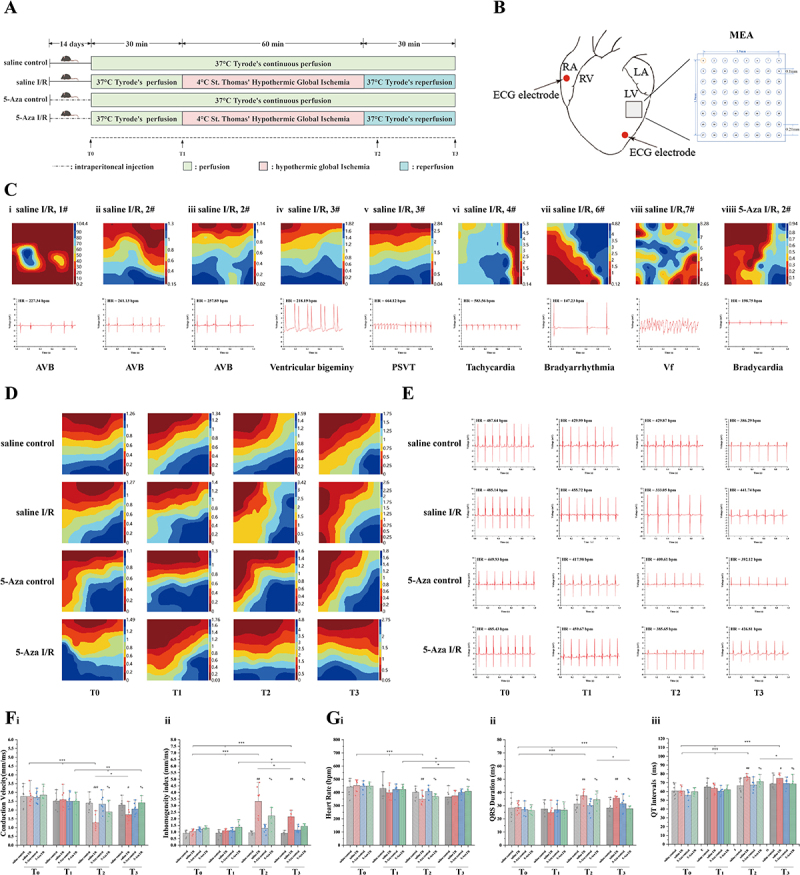
DNMT inhibition reduces reperfusion arrhythmias.

Control groups maintained stable sinus rhythm throughout the experimental protocol without observable arrhythmic events. Following reperfusion, saline I/R hearts demonstrated significant electrophysiological instability, requiring 6.2 ± 2.9 s for rhythm resumption (Table 1). Six of eight hearts developed various arrhythmias including atrioventricular blocks, ventricular bigeminy, paroxysmal supraventricular tachycardia, tachycardia, and bradyarrhythmias (Figure 1(C) i-viii). Notably, two hearts experienced fatal arrhythmic episodes characterized by severe bradycardia and ventricular fibrillation.

(A). Experimental protocol illustrating treatment groups (saline control, saline I/R, 5-Aza control, 5-Aza I/R). (B). Schematic of electrode placement for ECG and MEA recordings. (C). Representative isochronal maps and ECG traces of arrhythmic events observed in saline I/R hearts, including atrioventricular block (AVB), ventricular bigeminy, paroxysmal supraventricular tachycardia (PSVT), tachycardia, bradyarrhythmia, and ventricular fibrillation (VF). Only one transient bradycardia was observed in the 5-Aza I/R group. (D). Isochronal activation maps at different time points (T_0_–T_3_) across groups showing conduction slowing and heterogeneity in saline I/R hearts, which were alleviated by 5-Aza. (E). Representative ECGs at each time point demonstrating bradycardia and conduction interval prolongation in I/R hearts compared with controls. (F). Quantified conduction velocity (i) and conduction heterogeneity index (ii). (G). Quantified ECG parameters: heart rate (i), QRS duration (ii), and QT interval (iii). Data are presented as mean ± SD (*n* = 8 per group). Statistical analyses were performed using one-way ANOVA followed by Tukey’s post hoc test for continuous variables, and Fisher’s exact test for categorical variables. *indicates intragroup comparisons at different time points (vs. T_0_or T_1_), *p < 0.05, **p < 0.01 and ***p < 0.001; # indicates intergroup comparisons with the saline control group at the same time point,^#^*p* < 0.05,^##^*p* < 0.01; % indicates intergroup comparisons with the saline I/R group at the same time point,^%^*p* < 0.05. *p* value < 0.05 was considered statistically significant.

**Table 1.** Effects of 5-Aza on reperfusion arrhythmias in mice.

	Saline		5-Aza	
	Control	I/R	Control	I/R
Weight (g, $\overset{ˉ}{\chi }$±SD)	27.0 ± 0.8	27.1 ± 0.5	26.5 ± 0.9	26.8 ± 1.0
Arrhythmias occurrence (n/n, %)	0/8, 0%	6/8, 75%**	0/8, 0%	1/8, 25%^#^
Fatal arrhythmias occurrence among arrhythmic hearts (n/n, %)	0/0, 0%	2/6, 33.3%*	0/0, 0%	0/1, 0%
Time to rhythm resumption (s, $\overset{ˉ}{\chi }$±SD)	N/A	6.2 ± 2.9	N/A	5.9 ± 2.9

Notes: Data are expressed as mean ± SD from 8 mouse hearts per group. Continuous variables were analyzed using one-way ANOVA followed by Tukey’s post hoc test, and categorical variables (arrhythmia and fatal arrhythmia incidence) were compared using Fisher’s exact test. Comparisons within groups are indicated as *p < 0.05, **p < 0.01, ***p < 0.001; comparisons among groups are indicated as vs. Saline, ^#^*p* < 0.05, ^##^*p* < 0.01, ^###^*p* < 0.001. Statistical significance was defined as *p* < 0.05.

MEA and ECG analyses further demonstrated pronounced conduction abnormalities during reperfusion. Compared with the pre-ischaemic time point (T1), CV in saline I/R hearts decreased from 2.59 ± 0.49 to 1.28 ± 0.39 at T2 and remained reduced at 1.74 ± 0.40 at T3. In parallel, conduction heterogeneity increased markedly, with the conduction heterogeneity index rising from 1.11 ± 0.12 at T1 to 3.33 ± 1.12 at T2 and remaining elevated at 2.16 ± 0.31 at T3. QRS duration was also prolonged from 24.68 ± 5.55 at T1 to 37.45 ± 4.20 at T2 and 35.76 ± 4.05 at T3, while QT interval increased from 63.41 ± 5.88 at T1 to 76.19 ± 2.54 and 74.99 ± 5.24 at T2 and T3, respectively (Figure 1(D–G)). Heart rate was reduced during reperfusion, although this parameter showed greater variability than the other electrophysiological indices.

In contrast, 5-Aza I/R hearts showed comparable rhythm recovery time (5.9 ± 2.9 s) but demonstrated markedly improved arrhythmic profiles. Only one heart developed transient bradycardia that spontaneously resolved within 20 s, and no fatal arrhythmias occurred (Figure 1(C) ix). Electrophysiological deterioration was also attenuated by 5-Aza pretreatment. CV was higher in 5-Aza I/R hearts than in saline I/R hearts at both T2 (1.90 ± 0.41 vs. 1.28 ± 0.39) and T3 (2.42 ± 0.32 vs. 1.74 ± 0.40). Because baseline CHI showed mild intergroup variation, changes relative to T1 were further examined; the increase in CHI was smaller in 5-Aza I/R hearts than in saline I/R hearts at both T2 and T3. Consistent with this, absolute CHI values were also lower in 5-Aza I/R hearts at T2 (2.24 ± 0.67 vs. 3.33 ± 1.12) and T3 (1.43 ± 0.18 vs. 2.16 ± 0.31). QRS prolongation was reduced, particularly at T3 (27.64 ± 5.02 vs. 35.76 ± 4.05 in saline I/R), whereas QT prolongation showed partial attenuation at the early reperfusion time point. Collectively, these findings indicate that I/R induced a characteristic electrophysiological phenotype consisting of slowed conduction, increased conduction heterogeneity, and ECG interval prolongation, whereas 5-Aza pretreatment partially preserved electrical stability during reperfusion.

### DNMT inhibition alleviates MIRI and apoptosis while restoring Cx43 expression and localization

**Figure 2.**
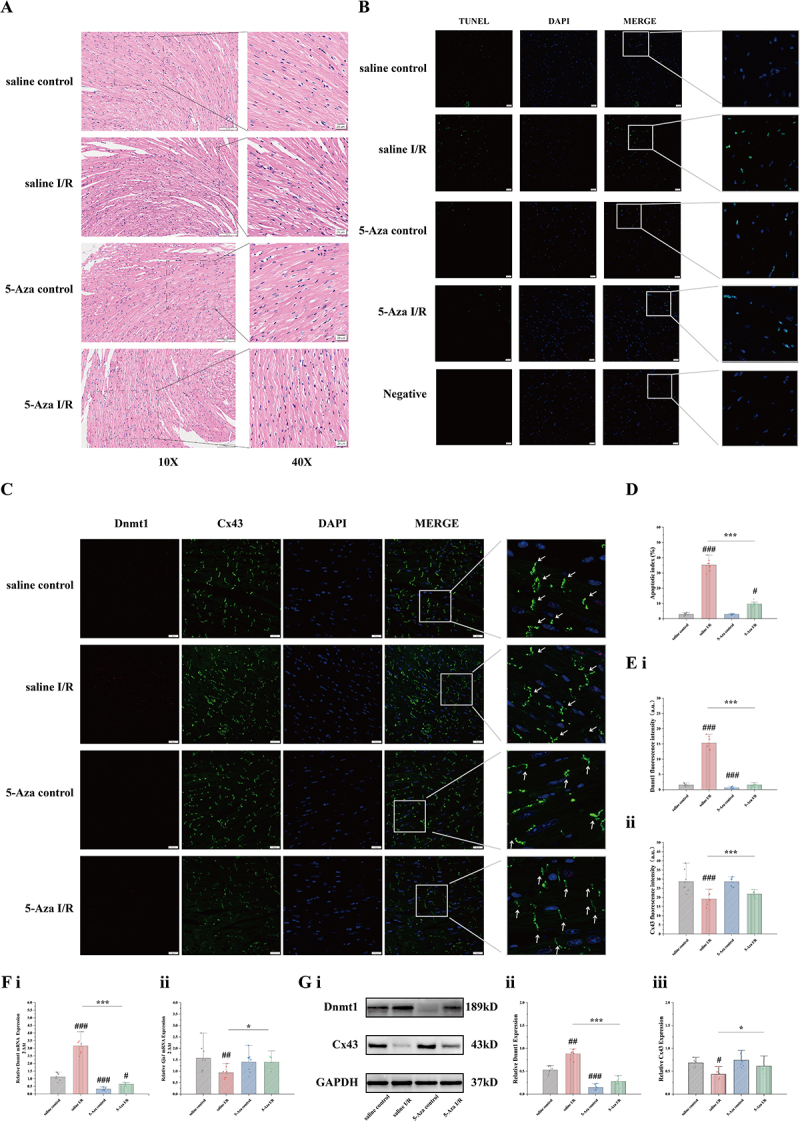
DNMT inhibition alleviates MIRI, reduces apoptosis, and restores Cx43 expression following I/R.

Histological analysis showed that I/R caused extensive myocardial damage, whereas DNMT inhibition offered marked tissue protection. HE staining showed relatively preserved myocardial architecture in the saline control and 5-Aza control hearts with organized fiber alignment and normal cellular morphology. In contrast, saline I/R hearts displayed expanded interstitial spaces, myocardial fibre disorganisation, focal fibre separation or fragmentation, and structural loosening consistent with acute tissue injury. These histological abnormalities were attenuated in the 5-Aza I/R group, which showed relatively better preservation of myocardial architecture and less interstitial widening and fibre disarray (Figure 2(A)). TUNEL staining revealed minimal apoptosis in controls but widespread cell death after I/R. 5-Aza substantially reduced apoptotic nuclei versus untreated I/R hearts (Figure 2(B,D)). Cx43 was concentrated at intercalated discs in control hearts, with sparse nuclear Dnmt1. I/R caused pronounced nuclear Dnmt1 accumulation and Cx43 loss from intercalated discs, with redistribution to lateral membranes. 5-Aza prevented Dnmt1 nuclear translocation and maintained Cx43 at intercalated discs (Figure 2(C,E)). qRT-PCR showed Dnmt1 increase and *Gja1* reduction after I/R. Western blots confirmed elevated Dnmt1 protein and decreased Cx43. 5-Aza normalized both proteins. DNMT inhibition was associated with attenuated MIRI and preservation of Cx43 expression and localization (Figure 2(F,G)).

(A). Representative HE staining images (10× and 40×) showing preserved myocardial architecture in saline control and 5-Aza control groups, widened interstitial spaces and myocardial fibre disorganisation in saline I/R hearts, and attenuation of these histological abnormalities in 5-Aza I/R hearts. (B). TUNEL staining (40×) demonstrating low apoptotic activity in control and 5-Aza control groups, markedly increased apoptosis in saline I/R hearts, and reduced apoptosis following 5-Aza treatment. (C). Immunofluorescence analysis of Dnmt1 and Cx43 expression and localization (60×). Saline I/R hearts exhibited increased nuclear Dnmt1 and disrupted, diffuse Cx43 distribution, whereas 5-Aza administration reduced nuclear Dnmt1 and restored Cx43 localization at intercalated discs. Dnmt1: CY3 (red); Cx43: FITC (green); nuclei: DAPI (blue). (D). Quantified analyses of TUNEL-positive nuclei. (E). Quantification of fluorescence intensity: (i) Dnmt1, (ii) Cx43. (F). Quantitative RT-PCR analysis of (i) Dnmt1 and (ii) Cx43 mRNA expression. (G). Western blot analysis of Dnmt1 and Cx43 protein expression (i) with densitometric quantification (ii, iii), normalized to GAPDH. Data are expressed as mean ± SD; #*p* < 0.05, ##*p* < 0.01, ###*p* < 0.001 vs. saline control; *p < 0.05, **p < 0.01, ***p < 0.001 vs. saline I/R. *p* value < 0.05 was considered statistically significant.

### *Heterogeneous methylation patterns within the* Gja1 *promoter after I/R*

**Figure 3.**
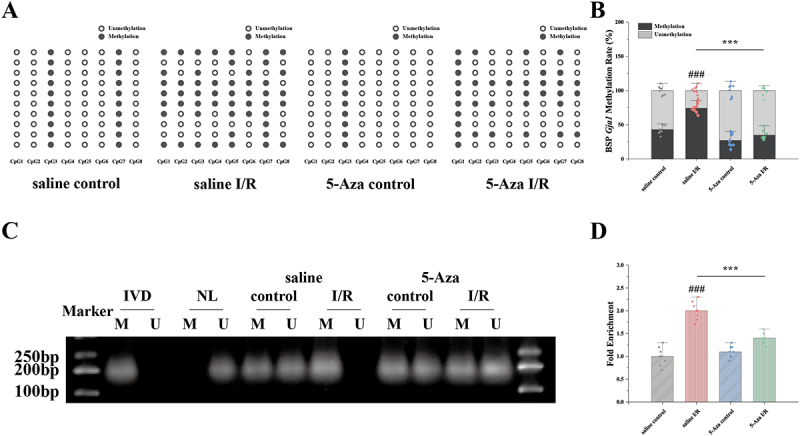
I/R is associated with CpG-resolved methylation changes and increased Dnmt1 enrichment at the *Gja1* promoter region.

**Figure 4.**
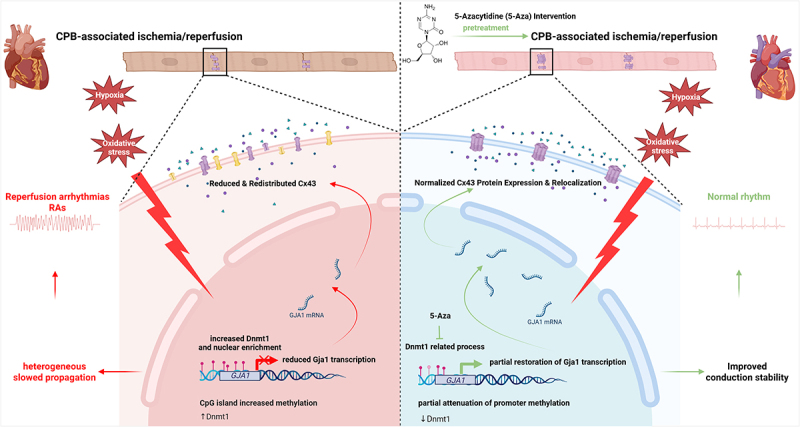
Epigenetic regulation of Cx43 remodeling during MIRI.

To characterise the epigenetic regulation of *Gja1* at the promoter level, we assessed methylation within a 472 bp promoter fragment containing 8 CpG dinucleotides. Sanger sequencing data were processed with BiQ Analyzer to generate per-CpG lollipop diagrams illustrating the methylation status at individual positions in representative animals from each group promoter (Figure 3(A)). In saline control hearts, the promoter fragment exhibited a relatively restricted basal methylation pattern characterised by consistent methylation at a limited subset of CpG positions, with the majority of sites unmethylated across analysed clones. Following I/R, the per-CpG methylation landscape was substantially altered in multiple positions, most notably CpG1, CpG3, CpG5, and CpG6, showed appreciably higher proportions of methylated clones relative to controls. The overall methylation status varied among individual clones within the I/R group, reflecting the focal and spatially heterogeneous character of epigenetic remodelling within this promoter region. In 5-Aza treated animals, per-CpG methylation signals were generally attenuated compared with the saline I/R group, though residual variability across positions and between individual animals was observed. These clone-level data are therefore interpreted as position-resolved descriptive evidence supporting focal methylation changes within the examined *Gja1* promoter region, complementary to the group-level quantitative analyses described below. At the group level, BSP summary was consistent with the overall *Gja1* promoter methylation rate was significantly elevated in saline I/R hearts relative to saline controls, and that 5-Aza pretreatment substantially attenuated this increase (Figure 3(B)). Complementing these findings, MSP confirmed these findings: the I/R group exhibited stronger methylated (M) bands and weaker unmethylated (U) bands, while 5-Aza treatment attenuated the methylated signals under MIRI (Figure 3(C)). Furthermore, ChIP assays demonstrated that I/R significantly enhanced the enrichment of Dnmt1 at the *Gja1* promoter, whereas this enrichment was substantially reduced following 5-Aza treatment (Figure 3(D)). Collectively, the converging BSP, MSP, and ChIP evidence supports an association between I/R, increased Dnmt1 promoter occupancy, altered methylation signals at the *Gja1* locus, and reduced *Gja1* transcriptional output; however, uniform promoter-wide methylation remodelling across the entire fragment within the acute reperfusion window was not established by the present data. These integrated molecular and functional relationships are summarized schematically in Figure 4.

(A). Representative clone-level BSP lollipop plots showing the methylation status of individual CpG sites within the examined *Gja1* promoter fragment. Each row represents one sequenced TA clone, and each column represents one CpG site. Filled circles indicate methylated CpGs, whereas open circles indicate unmethylated CpGs. (B). Summary of BSP methylation and unmethylation rates across experimental groups. Saline I/R hearts exhibited a significant increase in methylation compared with controls, while 5-Aza treatment markedly reduced methylation and increased unmethylated CpG sites. (C). Representative MSP gel electrophoresis showing methylated (M) and unmethylated (U) products. Saline I/R hearts displayed stronger methylated bands and weaker unmethylated bands compared to controls, whereas 5-Aza treatment reversed these changes. IVD (in vitro methylated DNA) and NL (normal lymphocyte DNA) served as positive and negative controls, respectively. (D). ChIP-qPCR analysis revealed that Dnmt1 enrichment at the*Gja1*promoter was significantly increased in saline I/R hearts compared with controls, and reduced by 5-Aza treatment. Data are presented as mean ± SD (*n* = 8 per group). *p < 0.05, **p < 0.01, ***p < 0.001 vs. saline I/R; ^###^*p* < 0.001 vs. saline control.

The schematic illustrates the proposed regulatory pathway under two conditions. Left panel (I/R condition): CPB-associated I/R triggers hypoxia and oxidative stress, which are associated with increased Dnmt1 expression and nuclear enrichment. Dnmt1 recruitment to the *GJA1* promoter CpG island leads to increased CpG methylation and transcriptional repression of *GJA1*, resulting in reduced *GJA1* mRNA levels. Consequently, Cx43 protein expression is decreased and its subcellular localization is disrupted, with redistribution away from intercalated discs to lateral membranes. These changes impair gap junction coupling, producing heterogeneous and slowed electrical propagation and ultimately contributing to reperfusion arrhythmias (RAs). Right panel (5-Aza intervention): Pretreatment with 5-azacytidine (5-Aza), a DNA methyltransferase inhibitor, attenuates Dnmt1-related promoter methylation at the *GJA1* locus, enabling partial restoration of *GJA1* transcription. This is associated with normalized Cx43 protein expression and relocalization to intercalated discs, improved gap junction integrity, more uniform electrical propagation, and enhanced conduction stability. Arrows indicate associative regulatory relationships consistent with the experimental observations; they do not imply direct or exclusive causality. The schematic was created using BioRender.

## Discussion

The present study presents evidence consistent with an epigenetic mechanism linking MIRI to the altered *Gja1* promoter methylation, remodeling of Cx43 and subsequent electrical instability. Using a Langendorff-perfused mouse heart model, we show that I/R injury is associated with the upregulation and nuclear accumulation of Dnmt1, concurrent with its recruitment to the promoter region of *Gja1*. These changes coincided with altered methylation signals within the examined *Gja1* promoter region and transcriptional repression of *Gja1*, and a reduction in Cx43 protein expression and its redistribution away from intercalated discs. At the functional level, these molecular alterations correlated with impaired conduction properties, characterized by reduced CV, prolonged QRS/QT intervals, and increased CHI. Importantly, inhibition of DNA methylation with 5-azacytidine partially restored Cx43 expression, and mitigated arrhythmogenesis. Collectively, these data support a model in which Dnmt1-associated acute stress-responsive epigenetic modulation of *Gja1* locus may contributes to the arrhythmogenic substrate in the reperfused heart, although establishing direct causality will require gene-specific epigenetic manipulation and higher-resolution temporal profiling of promoter methylation dynamics in future studies.

Cx43 is the predominant gap junction protein in ventricular cardiomyocytes, forming intercellular channels that enable rapid action potential propagation and synchronized contraction [21]. Normal cardiac electrophysiology critically depends on adequate expression, phosphorylation, and precise intercalated disc localization, ensuring efficient conduction across the ventricular myocardium [22]. Under I/R conditions, however, Cx43 undergoes maladaptive remodeling characterized by reduced protein expression [23–25], dephosphorylation of key C-terminal residues [26], and redistribution from intercalated discs to lateral cell borders (lateralization) [27]. These alterations compromise gap-junction integrity, weaken intercellular coupling, result in conduction slowing and increased spatial heterogeneity, which manifest as QRS prolongation and reduced CV [28]. Such disturbances create a substrate highly vulnerable to reentrant arrhythmias, a recognized mechanism underlying ventricular tachyarrhythmias and fibrillation [22]. Notably, Cx43 turns over with a half-life of approximately 1.3 h in the adult heart via proteasomal and lysosomal pathways [29]; accordingly, within the 60-min ischaemia and 30-min reperfusion protocol employed here, accelerated degradation combined with impaired synthesis and trafficking provides a kinetic basis for the detectable reduction in total Cx43 observed at the reperfusion endpoint. Recent evidence highlights that lateralized Cx43 does not merely reflect passive loss of gap junction coupling; rather, it assembles hyperactive hemichannels at the lateral sarcolemma that directly disrupt membrane excitability and perpetuate arrhythmogenesis through mechanisms independent of intercellular coupling defects [30]. The causal link between Cx43 dysfunction and RAs is well supported by interventional evidence: targeted overexpression of Cx43 enhances electrical coupling and suppresses arrhythmias [31], selective perturbation of its phosphorylation precipitates reperfusion-induced arrhythmias [25], and our group has demonstrated that EB1-dependent Cx43 trafficking [24], microtubule stabilisation during sevoflurane preconditioning [32], and chaperone support by STIP1 and HSP40/70/90 [33,34]. Collectively, these findings confirm that I/R-induced Cx43 remodeling, encompassing expression loss, post-translational modification and lateralization, directly underlies conduction disturbances and arrhythmia susceptibility, and they highlight the therapeutic potential of interventions targeting cytoskeletal dynamics, protein stability, or intercellular signaling.

DNMT1 is conventionally regarded as a maintenance methyltransferase that propagates CpG methylation during DNA replication [35]. However, a replication-independent chromatin recruitment has been described in stress-responsive mode: oxidative stress can trigger rapid enrichment of DNMT1 in chromatin-associated fractions and biases its localisation toward GC-rich promoter CpG islands [17], while protein interaction with the mismatch repair heterodimer MSH2–MSH6 facilitates this engagement and supports transcriptional dampening at CpG-island promoters without requiring replication forks [36]. In an ex vivo cardiac context, a Langendorff 30-min ischaemia/60-min reperfusion protocol has been reported to increase DNMT1 expression/activity together with global myocardial DNA methylation following reperfusion, supporting the feasibility of DNMT1–methylation changes within an acute I/R window comparable in scale to ours [18]. Importantly, transcriptional attenuation at CpG-rich regulatory regions does not necessarily require uniform methylation across an entire CpG island. In experimentally tractable systems, even a small number of methylated cytosines can suppress promoter output, with a threshold–type relationship between the number of methylated CpGs and the extent and spread of repression [37]. Consistent with the canonical repressive role of DNA methylation at gene regulatory regions [38], our data are consistent with this model, showing Dnmt1 upregulation and nuclear accumulation during I/R accompanied by increased methylation signals at the selected CpG sites within the *Gja1* promoter and Dnmt1 enrichment at this locus by ChIP. These findings provide an epigenetic mechanistic grounding for the observed reduction in Cx43 availability under reperfusion stress. These findings are consistent with independent reports that DNMT1 inhibition is associated with attenuated cardiac injury following I/R [39] and that aberrant DNMT1 activity has been implicated in the epigenetic suppression of cardioprotective gene programs under ischaemic stress.

Several limitations of this study should be acknowledged. First, arrhythmia outcomes were analyzed primarily as incidence-based categorical endpoints rather than arrhythmia burden (e.g., event frequency per unit time, duration, or sustained ventricular tachycardia/ventricular fibrillation (VT/VF) burden), which may underestimate more subtle electrophysiological differences between groups. Second, this study used a constant pressure Langendorff model to standardize perfusion conditions and to focus on acute electrophysiological and molecular changes after ischaemia and reperfusion. However, baseline in vivo cardiac function was not assessed by echocardiography before heart isolation. Therefore, although perfusion conditions were controlled during isolated heart experiments, pre-existing functional differences or systemic effects related to repeated 5-azacytidine exposure cannot be fully excluded. Third, although total Cx43 expression and localisation were examined, phosphorylated forms of Cx43 were not measured, and Cx43 associated with intercalated discs was not biochemically distinguished from redistributed membrane pools. As a result, the relative contribution of post-translational Cx43 remodelling to the observed conduction abnormalities remains unresolved. Fourth, BSP, MSP, and ChIP support altered methylation status within the examined *Gja1* promoter region, and clone-based BSP allowed per-CpG visualization of methylation patterns within the examined *Gja1* promoter fragment. These data should be interpreted as site-resolved descriptive support for focal promoter methylation heterogeneity rather than definitive temporal proof of rapid de novo methylation. Fifth, the mechanistic observations were derived from the isolated heart model without complementary in vitro hypoxia and reoxygenation experiments, which limits assessment of direct effects at the cellular level. Sixth, 5-azacytidine is a global modulator of DNA methylation with pleiotropic actions, and no specific manipulation of Dnmt1 or the *Gja1* locus was performed. The present findings should therefore be interpreted as pharmacological support for a mechanism involving DNA methylation rather than definitive proof of a Dnmt1 dependent causal pathway. Finally, only male mice were included in this study to maintain a relatively homogeneous experimental background, and the generalisability of these findings to females remains uncertain. Future studies incorporating more detailed arrhythmia quantification, baseline functional phenotyping, phospho Cx43 analysis, complementary in vitro validation, and inclusion of both sexes will help further define the specificity and translational relevance of this pathway.

## Conclusion

In conclusion, the present findings suggest that altered methylation signals within the examined *Gja1* promoter region may represent a mechanism linking epigenetic stress responses to gap junction remodeling and the development of RAs during MIRI. The multilevel convergence of Dnmt1 upregulation and promoter enrichment, site-specific CpG hypermethylation at the *Gja1* locus, reduced *Gja1* transcriptional output, and pharmacological reversal by 5-azacytidine collectively supports a Dnmt1-centred epigenetic mechanism as a candidate contributor to gap junction remodelling under acute reperfusion stress. Although further work is required to establish gene specific causality and to define the contribution of other regulatory mechanisms, the present findings support the view that acute epigenetic regulation may participate in gap junction remodelling during MIRI.

## Supplementary Material

Supplementary Table.docx

## Data Availability

All data generated and analyzed in this study, including raw data used for statistical analyses, are publicly available in the Zenodo repository at https://doi.org/10.5281/zenodo.18505509.
