# Supplementary material for: Dnmt1 associated Gja1 promoter methylation changes are implicated in Cx43 remodeling during acute myocardial ischemia/reperfusion injury
Source: Epigenetics. 2026 Jul 1;21(1):2694817. doi: 10.1080/15592294.2026.2694817 (PMC13336278; doi:10.1080/15592294.2026.2694817)
Supplement: Supplementary Table.docx [file KEPI_A_2694817_SM1528.docx]

**Supplementary Table 2.1 Primers used for BSP analysis of the *Gja1* promoter**

| Primer | Start | Size | Tm | GC% | C’s | Sequence (5'-3') |
| --- | --- | --- | --- | --- | --- | --- |
| Left | 1656 | 26 | 59.08 | 42.31 | 4 | GGAAATGTGATTTAAAAGGGATATTT |
| Right | 1868 | 26 | 58.25 | 27.6 | 9 | AAACAAACAAAAAAAACAACCTAATC |
| Product size: 213, Tm: 64.6, CpGs in product: 4 | | | | | | |
| Left | 1656 | 26 | 59.08 | 42.31 | 4 | GGAAATGTGATTTAAAAGGGATATTT |
| Right | 1869 | 27 | 58.31 | 55.56 | 9 | TAAACAAACAAAAAAAACAACCTAATC |
| Product size: 214, Tm: 64.6, CpGs in product: 4 | | | | | | |
| Left | 1656 | 26 | 59.08 | 42.31 | 4 | GGAAATGTGATTTAAAAGGGATATTT |
| Right | 1867 | 26 | 59.36 | 53.85 | 8 | AACAAACAAAAAAAACAACCTAATCA |
| Product size: 212, Tm: 64.6, CpGs in product: 4 | | | | | | |
| Left | 766 | 25 | 54.90 | 48.00 | 4 | TAGTTATTTTAGTGAGAGGGTGTTT |
| Right | 977 | 26 | 59.63 | 57.69 | 8 | ACAACTCCTTTAAAAATTCCCAAATA |
| Product size: 212, Tm: 64.6, CpGs in product: 7 | | | | | | |
| Left | 1655 | 27 | 59.81 | 40.74 | 4 | AGGAAATGTGATTTAAAAGGGATATTT |
| Right | 1868 | 26 | 58.25 | 27.6 | 9 | AAACAAACAAAAAAAACAACCTAATC |
| Product size: 214, Tm: 64.6, CpGs in product: 4 | | | | | | |

**Supplementary Table 2.2 Primers used for MSP analysis of the *Gja1* promoter**

| Primer | Start | Sequence (5'-3') |
| --- | --- | --- |
| Left M | 625 | TGGATATAAAGTTTTGAAAGTTCGA |
| Right M | 814 | TTCCTAAAAAACATTTTCCTACCG |
| Product size: 213, Tm: 64.9 | | |
| Left U | 625 | TGGATATAAAGTTTTGAAAGTTCGA |
| Right U | 815 | CTTCCTAAAAAACATTTTCCTACCA |
| Product size: 191, Tm: 64.1 | | |
| Left M | 625 | TGGATATAAAGTTTTGAAAGTTCGA |
| Right M | 814 | TTCCTAAAAAACATTTTCCTACCG |
| Product size: 190, Tm: 64.9 | | |
| Left U | 624 | TTGGATATAAAGTTTTGAAAGTTTGA |
| Right U | 815 | CTTCCTAAAAAACATTTTCCTACCA |
| Product size: 192, Tm: 64.0 | | |
| Left M | 625 | TGGATATAAAGTTTTGAAAGTTCGA |
| Right M | 814 | TTCCTAAAAAACATTTTCCTACCG |
| Product size: 190, Tm: 64.9 | | |
| Left U | 626 | GGATATAAAGTTTTGAAAGTTTGA |
| Right U | 815 | CTTCCTAAAAAACATTTTCCTACCA |
| Product size: 190, Tm: 64.1 | | |
| Left M | 624 | TTGGATATAAAGTTTTGAAAGTTTGA |
| Right M | 814 | TTCCTAAAAAACATTTTCCTACCG |
| Product size: 191, Tm: 64.9 | | |
| Left U | 625 | TGGATATAAAGTTTTGAAAGTTCGA |
| Right U | 815 | CTTCCTAAAAAACATTTTCCTACCA |
| Product size: 191, Tm: 64.1 | | |
| Left M | 625 | TGGATATAAAGTTTTGAAAGTTCGA |
| Right M | 813 | TCCTAAAAAACATTTTCCTACCG |
| Product size: 189, Tm: 65.0 | | |
| Left U | 625 | TGGATATAAAGTTTTGAAAGTTCGA |
| Right U | 815 | CTTCCTAAAAAACATTTTCCTACCA |
| Product size: 191, Tm: 64.1 | | |
